# Quantification of the evolution of firm size distributions due to mergers and acquisitions

**DOI:** 10.1371/journal.pone.0183627

**Published:** 2017-08-25

**Authors:** Sandro Claudio Lera, Didier Sornette

**Affiliations:** 1 ETH Zurich, Singapore-ETH Centre, Singapore, Singapore; 2 ETH Zurich, Department of Management, Technology, and Economics, Zurich, Switzerland; 3 Swiss Finance Institute, Geneva, Switzerland; East China University of Science and Technology, CHINA

## Abstract

The distribution of firm sizes is known to be heavy tailed. In order to account for this stylized fact, previous economic models have focused mainly on growth through investments in a company’s own operations (internal growth). Thereby, the impact of mergers and acquisitions (M&A) on the firm size (external growth) is often not taken into consideration, notwithstanding its potential large impact. In this article, we make a first step into accounting for M&A. Specifically, we describe the effect of mergers and acquisitions on the firm size distribution in terms of an integro-differential equation. This equation is subsequently solved both analytically and numerically for various initial conditions, which allows us to account for different observations of previous empirical studies. In particular, it rationalises shortcomings of past work by quantifying that mergers and acquisitions develop a significant influence on the firm size distribution only over time scales much longer than a few decades. This explains why M&A has apparently little impact on the firm size distributions in existing data sets. Our approach is very flexible and can be extended to account for other sources of external growth, thus contributing towards a holistic understanding of the distribution of firm sizes.

## 1 Introduction

In any established economy, companies of all sizes can be observed, ranging from small local businesses up to multinational corporations. The size of a company can conveniently be measured for instance in terms of yearly revenue, asset value, number of employees, and so on. It is by now a well-established fact that the distributions of firm sizes are heavy tailed [1, 2]. Knowledge of the firm size distribution is important because it has direct implications for an economy, for instance for monetary policy [3], employment [4] or innovation [5]. Generally, a heavy tailed firm size distribution implies that a large fraction of market impact is due to a few large firms that dominate the economy [6, 7]. It is then not surprising that the study of firm size distributions has received a great deal of attention both in the theoretical and empirical literature.

While theoretical models for the heavy tail nature of firm size distributions are manifold, at the heart of most explanations is the idea of proportional growth (Gibrat’s law [8]). Gibrat’s law states that the growth rate of a company is independent of its absolute size, or, equivalently, that the change in size is linearly proportional to its size. In absence of any other ingredient, the resulting firm size distribution is a log-normal function. It is noteworthy that, when the variance (in the log-variable) of the log-normal function is sufficiently large, it can appear close to a power law in an intermediate range of sizes [9–11]. Empirically however, the distribution of firms is fatter tailed than a log-normal function, and thus requires other ingredients than just proportional growth.

In addition to proportional growth, the various mathematical models have been enriched with other realistic ingredients such as firm heterogeneity [12–15], minimum firm size [16], births and deaths of firms [17–19], and sudden bankruptcy [20, 21]. Zipf’s law is then usually obtained due to subtle balance conditions between the growth rate and available external resources [16, 21, 22]. Finally, deviations from Gibrat’s fundamental assumption of proportional growth have also been considered [20, 23]. Sutton [24] gives a chronological wrap-up of the study of firm size distributions and a detailed overview on recent empirical studies is provided by Segarra and Teruel [25].

The theoretical models underlying the papers referenced above focus mainly on growth through investments in a company’s own operations (internal growth). The large impact of mergers and acquisitions (external growth) is most often not taken into account explicitly. For instance, over the period of 1995-1999, Schenk [26] observes that investments in acquisitions by North American and West European firms were approximately equal to sixty per cent of gross investments in machinery and equipment and they easily outpaced those in Research and Development (R&D). Investments in acquisitions were no less than about eight times higher than business enterprise expenditures on R&D. Although it must be mentioned that the period of 1995-1999 falls in the midst of a so-called merger wave [27, 28], these facts nevertheless make the point that mergers and acquisitions (M&A) are likely to be highly relevant for the study of firm size distributions. Taking into considerations M&A (as well as its counter-part, the spin-offs) constitutes an important component of a holistic understanding of the distribution of firm sizes.

In this article, we take a first step in this direction by formalizing the effect of M&A on the firm size distribution in terms of a non-linear partial integro-differential equation. This equation is formally equivalent to the coagulation equation from physics. By isolating the effect of M&A on the firm size distribution, we show that the coagulation equation allows to account for various empirical observations. Furthermore, our approach is very flexible and can conveniently be extended to also take into consideration spin-offs, internal growth, firm birth, bankruptcy and other growth related phenomena.

The remainder of this paper is structured as follows. In section 2, we summarize the literature that has studied the effect of mergers and acquisitions on firm size distributions. In section 3 and 4, we suggest a quantitative explanation for these observations in terms of the coagulation equation. Section 5 establishes a connection between the empirical findings and our theoretical calculations that let us account for the empirical stylized facts. Section 6 confirms further our approach by comparison with numerical solutions. Section 7 concludes and discusses possible extensions of the model.

## 2 The effect of mergers and acquisitions on Zipf’s law

While there is a large amount of literature examining Zipf’s law in general, there are only a few studies that investigate its relation to mergers and acquisitions. In this section, we extract the main conclusions from those studies formulated in terms of four key observations 1-4.

1. Examining a sample of large American firms, Ijiri & Simon [29] notice that smaller firms have a higher chance of being absorbed. Singh [30] concludes the same when studying firm sizes in the UK. Aaronovitch and Sawyer [31] confirm this finding by observing that UK firm sizes and probability of acquisitions are inversely related. We will see in the next section how this property has a very simple mathematical expression in terms of the coagulation equation.
2. Ijiri & Simon [32] examine the firm size distributions in 1956 and 1957 in a sample of large American firms. They conclude that the distribution of the 500 largest firms remained relatively unchanged from the effect of M&A. Their analysis thus supports the proposition that firm growth due to M&As would follow Gibrat’s law of proportional growth to the same extent as internal growth.
3. In a later study, Ijiri & Simon [29] examine the 831 largest US industrial firms in 1969 and conclude that the firm size distribution is clearly affected by M&A. Plotting the firm size distribution in a log-log plot after the M&A had taken place, they find that the distribution with M&As is shifted slightly to the right of the distribution without M&As. On top of that, both distributions are concave and not straight lines expected under Zipf’s law. In section 5, we will see how the difference between Ijiri & Simon’s study [29, 32] finds a natural explanation in terms of the coagulation equation.
4. Cefis et al. [33] examine the entire population of Dutch manufacturing firms. Unlike previous studies, they examine an entire population of firms and not only large firms. They conclude that M&As do not affect the size distribution when they consider the entire population of firms. Examining only firms that are at some point involved in an M&A, a shift of the firm size distribution towards larger sizes is noticed. This shift is not uniform but affects firms of different sizes in different ways. While the number of firms in the lower tail decreased, the number of firms in the central size classes increased.

A fifth observation is by Hannah and Kay [34]. Studying UK manufacturing firms in 1957, the authors decompose the growth rate into internal and external contributions and conclude that the observed power law coefficient is attributed to external growth. Furthermore, had it been just for internal growth, smaller firms would have grown faster than larger firms. These findings were later challenged and debated [35, 36]. This fifth observation addresses the interplay between internal and external growth. Albeit interesting and debated, accounting for this observation is not part of the current work and will not be further mentioned. However, as will become clear below, our model can be extended to also capture the mixed dynamics of external and internal growth.

In the subsequent sections, we set up a model that allows us to account for observations 1-4.

## 3 Mergers and acquisitions as a coagulation process

Let us denote by *p*(*m*, *t*) the distribution of firm sizes *m* at time *t*. We propose to model the effect of M&A on *p* as an equation that consists of two dynamic contributions: On the one hand, the distribution density at size *m* is increased due to the merger of a firm of size *m*′ with a firm of size *m* − *m*′ to a firm of size *m*. On the other hand, the density at size *m* is decreased through the merger of a company of size *m* with a company of size *m*′ to a company of size *m* + *m*′. Since this logic applies to all firm sizes *m*′, we have to take the integral (“sum”) over all such contributions, giving rise to the following integro-differential equation

$$\begin{array}{c} \frac{dp(m,t)}{dt}=\frac{1}{2}\int_{0}^{m}dm^{\prime }\;A\left(m^{\prime },m-m^{\prime }\right)p\left(m^{\prime },t\right)p\left(m-m^{\prime },t\right)-p\left(m,t\right)\int_{0}^{\infty }dm^{\prime }\;A\left(m,m^{\prime }\right)p\left(m^{\prime },t\right). \end{array}$$ (1)

We call *A*(*m*, *m*′) the merger kernel, describing the rate at which a firm of size *m* merges with a firm of size *m*′ to produce a firm of size *m* + *m*′ (we neglect here any dilution of size that may occur during the merger). The first term on the right-hand side of Eq (1) accounts for all the firms of size *m*′ that merge at time *t* with a company of size *m* − *m*′ to form a company of size *m*. Mergers of this type are weighted with the rate *A*(*m*′, *m* − *m*′) and then the ‘sum’ over all such *m*′, *m* − *m*′ pairs is taken. The factor 1/2 is to avoid double counting. The second term on the right hand side decreases the concentration of firms of size *m* by subtracting the sum of all firms of size *m* that merge at time *t* with a firm of size *m*′ to form a firm of size *m* + *m*′. We choose here a continuous description for mathematical convenience. Alternatively, the integrals in Eq (1) can be replaced by discrete sums. This does not qualitatively alter the presented results.

**Fig 1:**
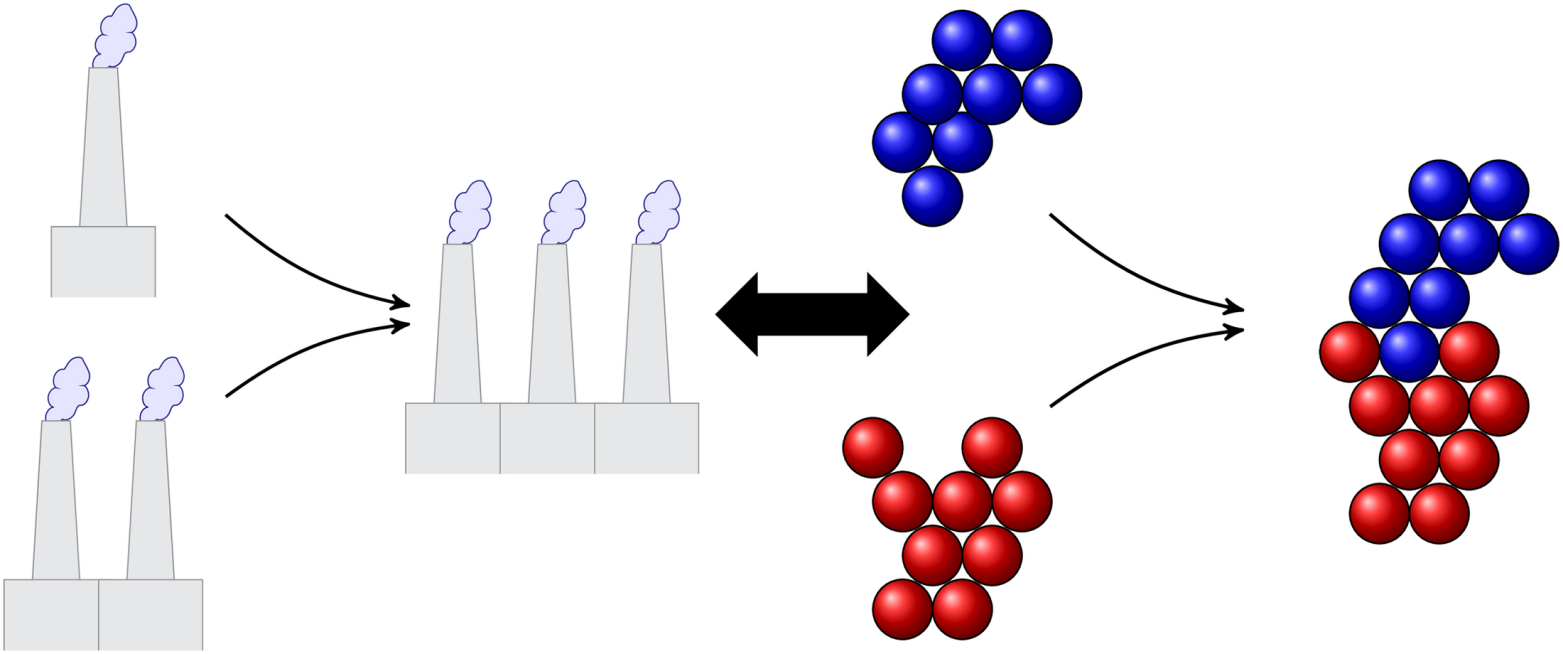
Both the merger and acquisition of firms (left side) and the coagulation of physical particles (right side) are formally described by the same mathematical Eq (1). The fact that the two processes are described by the same mathematical equation just reflects the generic process of growth by assembling.

As was pointed already out by Saichev et al. [20], Eq (1) is well-known in physics as Smulowski’s coagulation equation [37, 38]. This equation has been introduced to describe the evolution of the number density of particles with mass or size *m* as they consolidate (coagulation process). The coagulation equation has found many applications in astrophysics, cloud physics, polymer chemistry and formation of aerosols [39–41]. See Fig 1 for an illustration of this concept.

Considering the huge amount of literature in physics and mathematics that has been devoted to Eq (1), it should be convenient to fall back on one of the many known solutions. It turns out, however, that our merger equation is different from what is usually considered in physical systems. This is for two reasons. First, as is discussed in the next section, our merger kernel *A*(*m*, *m*′) is a decaying function in *m* and *m*′. Most physical systems assume that the coagulation kernel *A*(*m*, *m*′) be a (homogeneous and) increasing function in *m* and *m*′. Second, at time *t* = 0, we start from a heavy tailed firm size distribution. In physical applications, one usually starts from a thin tailed or even point-like initial distribution. This changes significantly the solution methods discussed in the literature. Only recently, power law initial distributions in combination with decaying coagulation kernels have been considered [42–45]. However, merely approximate scaling results in the limits of small and large masses are known and several issues are not yet clearly resolved. The state of the art from a mathematical perspective is well summarized by Costa [46]. We conclude that there are so far no known solutions to the merger equation that we develop and solve in the next section.

The coagulation Eq (1) is essentially just one of many potential growth mechanisms, which can be summarized in the following population-balance equation:

$$\frac{\partial p\left(m,t\right)}{\partial t}={\left[\frac{\partial p\left(m,t\right)}{\partial t}\right]}_{\text{M\&A}}+{\left[\frac{\partial p\left(m,t\right)}{\partial t}\right]}_{\text{spin-off}}+{\left[\frac{\partial p\left(m,t\right)}{\partial t}\right]}_{\text{int. growth}}+{\left[\frac{\partial p\left(m,t\right)}{\partial t}\right]}_{\text{exogenous}}.$$ (2)

The *M*&*A* component is given by Eq (1). The spin-off off dynamics obeys

$$\begin{array}{c} {\left[\frac{\partial p\left(m,t\right)}{\partial t}\right]}_{\text{spin-off}}=2\overset{\infty }{\underset{0}{\int \;}}\text{d}\tilde{s}\;K\left(m,\;\tilde{m}\right)p\left(m+\tilde{m}\right)-p\left(m,t\right)\overset{m}{\underset{0}{\int }}\text{ d}\tilde{m}\;K\left(m-\tilde{m},\tilde{m}\right) \end{array}$$ (3)

with *K* the spin-off kernel. The interpretation of Eq (3) is very similar to the one of Eq (1). The first term on the right hand side of Eq (3) captures the split of a firm of size $m+\tilde{m}$ into two firms of size *m* and $\tilde{m}$, respectively. The second term accounts for all the firms of size *m* that split into two smaller firms. Internal growth can be captured for instance through the Fokker-Planck equation

$$\begin{array}{c} {\left[\frac{\partial p\left(m,t\right)}{\partial t}\right]}_{\text{int. growth}}=-\frac{\partial \;}{\partial m}\left[\mu \left(m\right)\;p\left(m,t\right)\right]+\frac{1}{2}\frac{\partial^{2}\;}{\partial m^{2}}\left[\sigma {\left(m\right)}^{2}p\left(m,t\right)\right] \end{array}$$ (4)

with the functions *μ* and *σ* proportional to *m*, according to Gibrat’s law of proportional growth [8]. The exogenous growth term in Eq (2) captures the various additional influences such as sudden bankruptcy, creation of new firms etc.

Eq (2) can, in general, only be solved numerically, for which many parameters have to be fixed (see however Chap. 10 in [20], which offers an analytical solution in the case of non-proportional growth in the presence of M&A and spin-offs). Instead, here we isolate the perturbative effect of M&A on the firm size distribution, for which we can obtain an approximate analytical solution. This allows us to relate our results to previous empirical studies. We account (partially) for the existence of additional growth mechanisms by starting from an initial Zipf’s law distribution, that can be generated under various different conditions of internal growth, firm birth and firm bankruptcy [21]. This assumption is justified insofar as M&As have become especially prominent in the second half of the last century (cf. Fig 1 in [28]), whereas the observations of heavy tailed firm size distributions date back to Gibrat [8]. We thus study the perturbative effect of M&A on an already developed firm size distribution.

Finally, we can neglect spin-off activity, which occurs with a much smaller frequency that M&A’s, as illustrated by the Japanese firm database provided by the corporate research company TDB. For 2014, for instance, out of a total of roughly 1,100,000 firms, there was approximately 4,500 mergers and acquisitions and less than 100 spin-off events.

## 4 Coagulation equation for heavy tailed distributions: An approximate solution

The solution *p*(*m*, *t*) of Eq (1) describes the firm size distribution under the influence of M&A at time *t*. We assume that our observation starts at time *t* = 0 from a heavy tailed initial distribution. Here, time *t* is an abstract parameter that will be related to real time only in the next section. In this section, we give an analytical first order solution to Eq (1).

### 4.1 Specification of the merger kernel and initial distribution

The coagulation Eq (1) is not yet fully determined. We must also specify a merger kernel *A*(*m*, *m*′) and an initial distribution *p*(*m*, *t* = 0). The kernel *A*(*m*, *m*′) describes the rate at which a firm of size *m* merges with a firm of size *m*′ to form a firm of size *m* + *m*′. According to observation 1, this must be a decaying function in *m* and *m*′. A natural choice is thus

$$\begin{array}{c} A\left(m,m^{\prime }\right)=e^{-\alpha m}+e^{-\alpha m^{\prime }} \end{array}$$ (5)

for some *α* > 0. Clearly, other kernels are possible. In particular, our intuition tells us that a take-over becomes more likely with the acquiring firms becoming relatively more dominant in size. We will see below that there is no strong dependence of our results on the kernel.

As stated in the introduction, the exact shape of the firm size distributions is debated. We could thus choose either a log-normal or a Pareto as initial distribution. Since the log-normal is considerably more difficult to treat analytically, we decide to work here with a power law. Numerical results for the log-normal are found in section 6. Assuming a power law initial distribution means $p\left(m,t=0\right)=\mu m_{0}^{\mu }/m^{1+\mu }$ for some lower cut-off *m*_0_ and power-law exponent *μ*. Zipf’s law is recovered exactly by setting *μ* = 1. On the other hand, as already mentioned, the log-normal law with sufficiently large variance (in the log-variable) resembles a power law with *μ* ≳ 0 over a wide range of values [9–11]. Empirically, various values of *μ* have been reported, ranging from *μ* = 1/2 up to values close to *μ* = 2. Segarra and Teruel [25] provide a good overview.

Working with a power-law $p\left(m,t=0\right)=\mu m_{0}^{\mu }/m^{1+\mu }$ brings along two inconveniences. First, its Laplace transform involves the incomplete Gamma function that is analytically difficult to handle. Second, we must introduce an arbitrary lower cut-off *m*_0_. A way to avoid both of these problems is to work with the fractional exponential distribution (FED) [47] introduced by as a generalization of a Poisson process with long memory. For 0 < *μ* ≤ 1, the FED is of the form [48]

$$\begin{array}{c} f_{\mu }\left(m\right)=-\frac{d\;}{dm}E_{\mu }(-m^{\mu }), \end{array}$$ (6)

or, equivalently [49],

$$\begin{array}{c} f_{\mu }\left(m\right)=m^{\mu -1}E_{\mu ,\mu }(-m^{\mu }), \end{array}$$ (7)

with the Mittag-Leffler functions *E*_*μ*_ and *E*_*μ*,*ν*_ defined by

$$\begin{array}{c} E_{\mu }\left(z\right)=\sum_{n=0}^{\infty }\frac{z^{n}}{\Gamma (\mu n+1)}\;\;\;\;\;\;\text{and}\;\;\;\;\;\;E_{\mu ,\nu }\left(z\right)=\sum_{n=0}^{\infty }\frac{z^{n}}{\Gamma (\mu n+\nu )}. \end{array}$$ (8)

Repin and Saichev [48] show that *f*_*μ*_ is asymptotically a power law:

$$\begin{array}{c} f_{\mu }\left(m\right)\approx \{\begin{array}{cc} m^{\mu -1}/\Gamma \left(\mu \right), & m\to 0;\\ m^{-(1+\mu )}/\mu \Gamma \left(1-\mu \right), & m\to \infty . \end{array} \end{array}$$ (9)

What makes the FED extremely useful compared to a strict Pareto initial distribution—in fact, this is its defining property—is its simple Laplace transform,

$$\begin{array}{c} {\hat{f}}_{\mu }\left(k\right)=\int_{0}^{\infty }dm\;e^{-mk}f_{\mu }\left(m\right)=\frac{1}{1+k^{\mu }}. \end{array}$$ (10)

By working with *f*_*μ*_, we restrict ourselves to exponents *μ* ∈ (0, 1]. Once again, numerical results in section 6 will confirm that our results are not strongly dependent on the exact choice of *μ*.

### 4.2 First order analytical solution

There are only a few known analytical solutions to Eq (1). All of them involve a kernel *A* that is an increasing function in *m* and *m*′ and most explicit solutions consider initial distributions with finite support. Here we consider an exponentially decaying kernel and initial distribution *f*_*μ*_ with a power law tail. We derive first order analytical results that account for empirical observations already very well. The reader that is only interested in the final result can skip directly to the next section. A detailed step by step solution is available from the authors upon request.

We start by noting that kernel (5) is of the form *A*(*m*, *m*′) = *F*(*m*) + *F*(*m*′) for some function *F* (here *F*(*m*) = exp(−*αm*)). Hendriks et al. [50] have shown that kernels of this type allow for a convenient simplification of the coagulation equation. We introduce *M*_0_(*t*) the zeroth order moment of *p*, which is defined by

$$\begin{array}{c} M_{0}\left(t\right)\equiv \int_{0}^{\infty }dm\;p\left(m,t\right)\;. \end{array}$$ (11)

Note that *M*_0_(*t*) is in general not constant since it is related to the total number of firms as a function of time (see expression (22) below). The total number of firms can vary as a function of the combined effects of creation, bankruptcies, merger and acquisitions as well as spin-offs. Reporting the substitutions

$$\begin{array}{c} q\left(m,\theta \right)\equiv \frac{p(m,t)}{M_{0}\left(t\right)},\;\;\;\;\;\;d\theta =dt\;M_{0}\left(t\right),\;\;\;\;\;\;\theta \left(t=0\right)=0 \end{array}$$ (12)

into Eq (1) yields

$$\begin{array}{c} \frac{dq(m,\theta )}{d\theta }=[Fq*q]\left(m,\theta \right)-F\left(m\right)q\left(m,\theta \right) \end{array}$$ (13)

where ‘*’ denotes the convolution operator. We interpret *q*(*m*, *θ*) as a rescaled version of *q*(*m*, *t*) with rescaled time parameter *θ*. Integro-differential equations such as Eq (13) are best solved in Laplace space, where the convolution turns into a multiplication. We then take the Laplace transform of Eq (13) with *F*(*m*) = exp(−*αm*) and obtain

$$\begin{array}{c} \frac{d\hat{q}\left(k,\theta \right)}{d\theta }=[\hat{q}\left(k,\theta \right)-1]\hat{q}\left(k+\alpha ,\theta \right) \end{array}$$ (14)

where $\hat{q}\left(k\right)$ is the Laplace transform of *q*(*m*). Eq (14) is a mixed functional differential equation [51]. Analytical solutions to non-linear equations of this type are not common. Instead, we make use of the fact that *α* must be small. The coagulation kernel describes the rate at which a company of size *m* merges with a firm of size *m*′. Since mergers are observed also between large firms (as measured for instance in terms of revenue or number of employees), kernel (5) must assign non-vanishing probability also to events with *m*, *m*′ ≫ 1. Consequently, *α* ≪ 1 and we can approximate $\hat{q}\left(k+\alpha \right)$ with a first order Taylor expansion, $\hat{q}\left(k\right)+\alpha {\hat{q}}^{\prime }\left(k\right)$.

Plugging this approximation into Eq (14) results in

$$\begin{array}{c} \frac{\partial \hat{q}\left(k,\theta \right)}{\partial \theta }+\alpha \left(1-\hat{q}\right)\frac{\partial \hat{q}}{\partial k}=\hat{q}\left(\hat{q}-1\right). \end{array}$$ (15)

This is a quasi-linear first order partial differential equation and can be solved analytically with the method of characteristics. Its solution, together with initial condition (10), reads $\hat{q}\left(k,\theta \right)={\left(1+k_{0}^{\mu }\left(k,\theta \right)e^{\theta }\right)}^{-1}$ where *k*_0_(*k*, *θ*) is the inversion of

$$\begin{array}{c} k\left(k_{0},\theta \right)=\alpha \log(\frac{1+k_{0}^{\mu }e^{\theta }}{1+k_{0}^{\mu }})+k_{0}. \end{array}$$ (16)

It is not difficult to see that for the entire range of realistic values of *k*, *α* and *θ* < 1, the approximation *k* ≈ *k*_0_ holds very well (see below for an explanation why *θ* < 1). We conclude

$$\begin{array}{c} \hat{q}\left(k,\theta \right)=\frac{1}{1+k^{\mu }e^{\theta }}. \end{array}$$ (17)

Comparing with Eq (10), the inverse Laplace transform of $\hat{q}\left(k,\theta \right)$ is just a rescaled FED,

$$\begin{array}{c} q\left(m,\theta \right)=e^{-\theta }m^{\mu -1}E_{\mu ,\mu }(-e^{-\theta }m^{\mu }). \end{array}$$ (18)

Finally, we have to transform back to real time *t*. Integrating the second equation of Eq (12) gives $\theta =\int_{0}^{t}d\tau \;M_{0}\left(\tau \right)$. The zeroth moment *M*_0_ is nothing but $\hat{p}\left(k=0,t\right)$. Taking the Laplace transform of Eq (5) with *F*(*m*) = exp(−*αm*) and setting *k* = 0 thus results in the following equation for *M*_0_,

$$\begin{array}{c} \frac{dM_{0}}{dt}=-\hat{p}\left(\alpha ,t\right)M_{0}=-\hat{q}\left(\alpha ,\theta \left(t\right)\right)M_{0}^{2}=-\frac{M_{0}^{2}}{1+\alpha^{\mu }e^{\theta }}. \end{array}$$ (19)

This last equation involves $\theta =\int_{0}^{t}d\tau \;M_{0}\left(\tau \right)$. To get rid of this dependency, we self-consistently set $\theta \left(t\right)=\theta^{\prime }\left(0\right)t+O(t^{2})\approx t$ in Eq (19). Of course, this assumption has to be justified through a posteriori sanity checks. Formally, we can appeal to the standard formal approach in applied mathematics. Consider a problem *P*, whose set of solutions is {*S*}. Suppose that we can approximate *P* by *P*_*a*_ and this approximation is valid only for those solutions in {*S*} that belong to a certain domain *V* ⊂ {*S*}. Let us consider the set of all solutions {*S*_*a*_} of problem *P*_*a*_. If these solutions {*S*_*a*_} belong to the domain *V*, then the solutions {*S*_*a*_} approximate the true solutions {*S*} of the initial full problem *P*. In fact, as will become visible below, our approximation only induces a slight overestimation of *θ*(*t*), leading at most to an understatement of our main result. Eq (19) is now solved with separation of variables, giving

$$\begin{array}{c} M_{0}\left(t\right)={\left(1+t+\text{log}\left(\frac{1+\alpha^{\mu }}{1+\alpha^{\mu }e^{t}}\right)\right)}^{-1}=1-\frac{t}{1+\alpha^{\mu }}+O\left(t^{2}\right), \end{array}$$ (20)

and then,

$$\begin{array}{c} \theta =\int_{0}^{t}d\tau \;M_{0}\left(\tau \right)=t-\frac{t^{2}}{2(1+\alpha^{\mu })}+O(t^{3}). \end{array}$$ (21)

**Fig 2:**
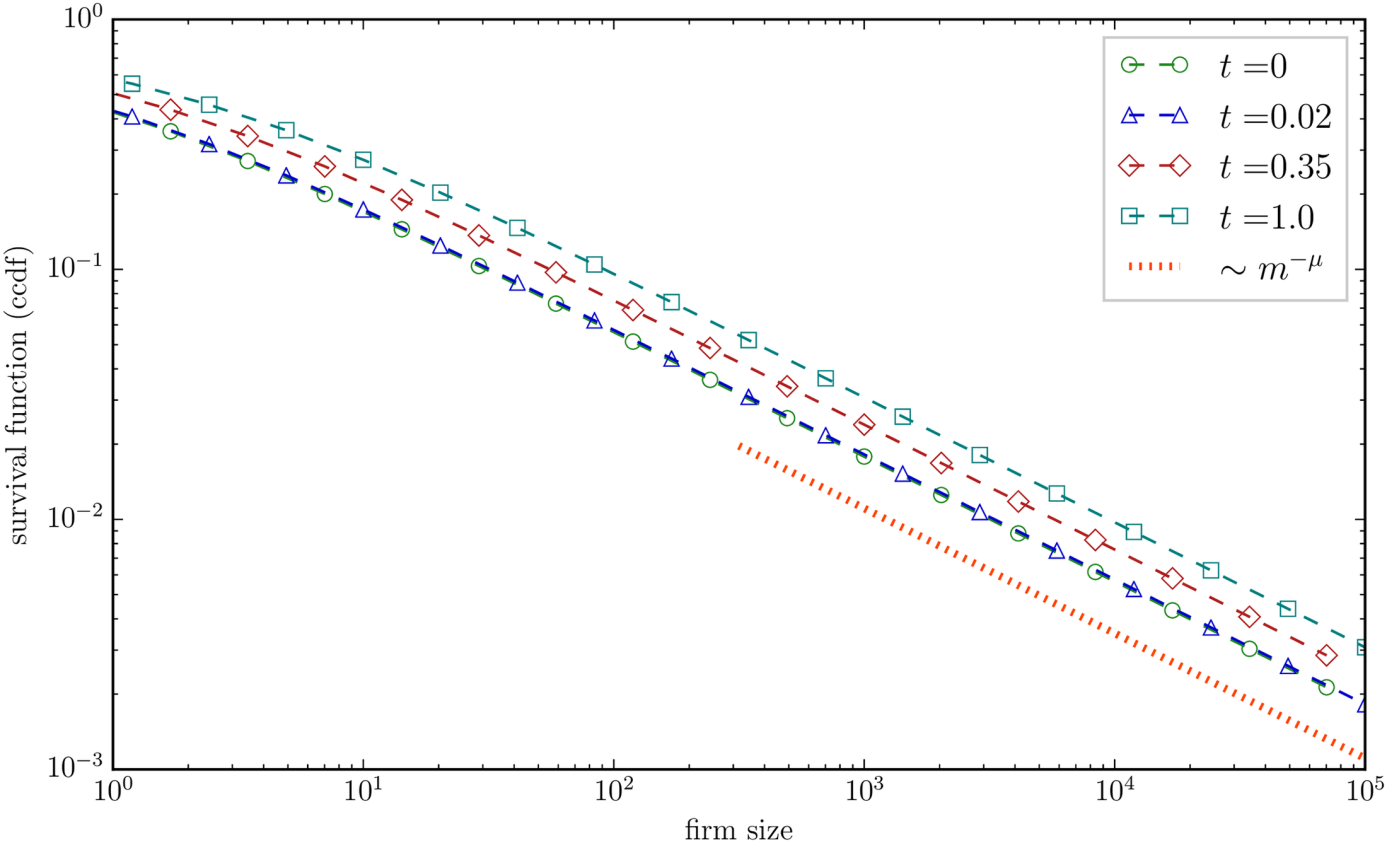
The first order solution of the coagulation Eq (1) with exponential kernel (5) and initial distribution Eq (7) is given by Eq (18) with *θ*(*t*) determined via Eq (21). Here, we show the corresponding survival function (or complementary cumulative distribution function) $\text{ccdf}\left(m\right)\equiv 1-\int_{0}^{m}dm^{\prime }\;p\left(m^{\prime }\right)$ for *μ* = 0.5 and *α* = 0.01. The dependence on *α* is extremely weak, which is why we do not show different choices. The times *t* are chosen in correspondence to the dataset considered by Cefis et al. [33]. All distributions are normalized to one at all times *t*.

In conclusion, the first order solution to Eq (1) is given by Eq (18) with *θ*(*t*) determined via Eq (21). This solution is depicted in Fig 2 for *μ* = 0.5 at different times *t*. We notice that, for times *t* < 1, only mild deviations from the initial distribution are observed. This is in line with observations 2–4, as we show in the next section.

## 5 Connecting the coagulation equation to empirical data

The first order solution to Eq (1) was derived in the previous section as Eq (18) with *θ*(*t*) determined by Eq (21). So far, we have treated time *t* as an abstract parameter. The connection to real time is established by noting that the coagulation kernel *A*(*m*, *m*′) is actually a rate.

Denote the number of firms at time *t* by *N*(*t*). It holds that

$$\begin{array}{c} N\left(t\right)=N_{0}\int_{0}^{\infty }dm\;p\left(m,t\right)=N_{0}M_{0}\left(t\right) \end{array}$$ (22)

with *N*_0_ ≡ *N*(*t* = 0). Then, the total number of firms that have ‘vanished’ through M&A since time *t* = 0 is given by

$$\begin{array}{c} \Delta N\left(t\right)=N_{0}-N\left(t\right)=N_{0}(1-M_{0}\left(t\right)). \end{array}$$ (23)

A priori, Δ*N* < 0 is also possible, meaning that newly born firms and spin offs outweigh the mergers and increase the total number of firms in an economy. The pure coagulation equation cannot account for such an observation. Here, we do not need to consider this case since 2–4 all report Δ*N* > 0. However, it is, at least in principle, straightforward to extend Eq (1) to the inclusion of spin-offs and firm births (cf. equation (10.5) in [20]).

Knowing *M*_0_(*t*), Eq (23) allows us to solve for time *t* as a function of observed Δ*N*. In our case, we deduce with Eq (20) that

$$\begin{array}{c} t=(1+\alpha^{\mu })\frac{\Delta N(t)}{N_{0}}\approx \frac{\Delta N(t)}{N_{0}}. \end{array}$$ (24)

In this last approximation, we have used that *α* ≪ 1. This is justified for the empirical studies below, for which mergers between large companies are reported. We have thus systematically shown that there is negligible dependence on *α*, meaning that the kernel *A* can be approximated to first order as constant for small times. This result can be made intuitive when noticing that, even for a constant kernel, the far right tail of the distribution is only affected for long times (see also numerical solution in section 6). For small times, it is mostly the lower part of the distribution that is affected by coagulation. For small *m*, exp(−*αm*) ≈ 1 is a good approximation.

Let us now turn to observations 2–4. The most thorough study was conducted by Cefis et al. [33]. They study a comprehensive data set of roughly 60,000 Dutch manufacturing firms including firm entries, exits, spin-offs, mergers and acquisitions. Measuring firm size in number of employees and accounting exactly for all relevant events, Cefis et al. [33] estimate both the firm size distribution in the beginning and in the end of the year 1997. The starting distribution is found to be best fitted by a log-normal. When plotting the starting and final distribution in one figure, it is seen that the starting and final distribution largely overlap, thus demonstrating that the firm size distribution seems to be unaffected by M&A (cf. Fig 2 there). Since 1997 was a year of high M&A activity of Dutch firms, this observation seems puzzling. While Cefis et al. [33] suggest a number of reasons, this observation finds a natural explanation within our framework. During 1997, 3,899 firms were involved in activities related to M&A and their spin-offs and divestures. At the end of the year, 2,564 remained, which suggests that M&As, spin-offs and divestitures have decreased the numbers of firms active in the manufacturing sector by Δ*N* = 1,335. With *N*_0_ = 57,329, we predict from Eq (24) a temporal evolution of merely *t* ≈ 0.02. As is visible in Fig 2, the *p*(*m*, *t* = 0.02) curve is hardly distinguishable from the *p*(*m*, *t* = 0) curve. The parameter *t* is related to real time (≈365 days) via a rate, namely the rate of M&A events. The contribution of our analysis is thus to quantify that the M&A events over the full year of 1997 amount in aggregate to a tiny perturbation to the initial firm distribution.

In a next step, in order to emphasize the possible effects of M&As, Cefis et al. [33] restrict their dataset to the firms that are directly involved in a M&A event during the year 1997. The result is a final distribution that is slightly above the starting distribution, showing that the M&As did have an effect on the distribution (cf. Fig 4 there). Again, their result is in good correspondence with our theory. Having *N*_0_ = 3,899 firms in the beginning that will undergo a M&A event and 2,564 of these firms at the end of 1997, we calculate Δ*N* = 1,335. According to Eq (24) this corresponds to *t* = 0.35. In our model, this results in a distribution that lies also slightly above the initial distribution, as shown in Fig 2.

In a very similar fashion, we can explain the observations of Ijiri & Simon [29, 32], at least semi-quantitatively. The major difference between their datasets and the one considered by Cefis et al. [33] is that Ijiri & Simon [29, 32] consider only the tail of the distribution, and do not have access to a complete dataset. In their first study, Ijiri & Simon [32] consider the *N*_0_ = 500 largest industrial firms (in terms of sales volume) and the number of firms that they have bought. They do not find any deviation from Zipf’s law through M&A. In our model, this is very natural. Only a total of Δ*N* = 19 net mergers are observed, resulting in a small time value of *t* ≈ 0.038. It is then clear from our calculations that no deviation from Zipf’s law is to be expected.

**Fig 3:**
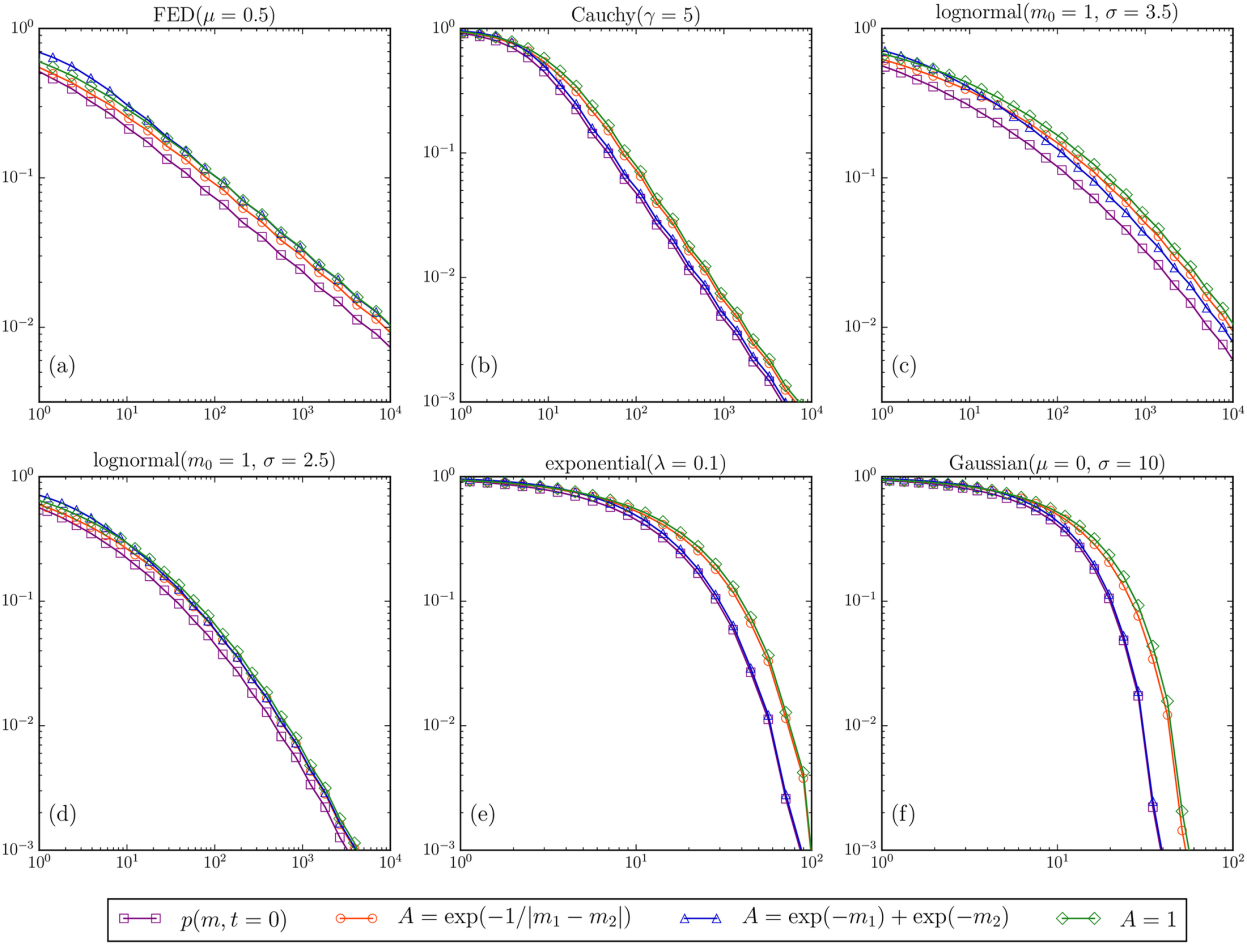
Numerical solution, for the complementary cumulative distribution functions (ccdf) of firm sizes, of the coagulation Eq (1) at time *t* = 1 for different initial distributions. All distributions are normalized to one at all times *t*. We show three different kernels: constant, decaying in company size, and increasing with the difference in company size. Fig (a) corresponds to the initial distribution being a fractional exponential distribution with asymptotic Pareto tail with exponent *μ* = 0.5. We can see that even for the exponentially decaying kernel deviations from the constant kernel solution are small, thus justifying our previous approximations. Similarly, Fig (b) corresponds to the initial distribution being the heavy-tailed Cauchy distribution with shape parameter *γ* = 5 and asymptotic Pareto tail *μ* = 1 (Zipf’s law). Fig (c) and (d) correspond to the initial distribution being a log-normal law with different values of *σ* (see definition (25)). This confirms that, over a finite size range and for times up to *t* = 1, the behavior of the firm distribution as a function of time for the log-normal initial distribution is similar to the case of the power law distributions. Finally, Fig (e) and (f) exemplify that, for light-tailed initial distributions, the dependence on the kernel can appear more pronounced.

In a follow-up paper, Ijiri & Simon [29] consider a much larger data set and come to the conclusion that M&A does change the firm size distribution by shifting it slightly above from what we would expect without mergers. They do not have a complete dataset, so they provide an estimate. It is estimated that the 831 largest industrial firms in 1969 would actually be 1,002 companies, if it had not been for a large amount of mergers in the preceding decade. Based on their estimate, we determine *N*_0_ = 1,002 and Δ*N* = 1,002 − 831 = 171, and thus *t* = 0.17. The effect that they observe is that the actual curve is shifted slightly to the right of the hypothetical curve without mergers (cf. Fig 1 there). This is again in agreement with what we would expect from our model with *t* = 0.17. Furthermore, when plotted in a double logarithmic plot, their observed distributions are concave and thus clearly different from a straight Pareto line, suggesting the lognormal distribution could provide a good fit. That this has no influence on our result becomes clear in the next section, especially from Fig 3(c) and 3(d).

In conclusion, our model accounts for observations 2–4. Furthermore, we can see that, in order to observe significant deviations from the initial distribution, a large number of mergers and acquisitions (of the order of the number of firms in the dataset) need to be observed, potentially over a long time horizon. This can be traced back to the fact that the heavy tail of the distribution is asymptotically unaffected by the coagulation process with decaying or constant merger kernel. If the firm size distribution was light tailed, deviations in the right end of the distribution would become apparent at an earlier stage, i.e. with fewer M&A observations. See also Fig 3(e) and 3(f).

## 6 Generality of our results

In the previous sections, we have presented a first order analytical solution to the coagulation equation. Thereby, several assumptions and approximations have been made for mathematical convenience. In this section, we show that our results apply more broadly and are not dependent on the assumptions made. Concretely, we use a second-order Runge-Kutta method with variable time step [52] to solve the coagulation Eq (1) numerically.

Observation 1 states that smaller firms have a higher chance of being absorbed. We have thus assumed an exponentially decaying merger kernel. While this is a natural choice, other shapes are possible. For instance, we could imagine a Pareto kernel *A*(*m*, *m*′) = 1/*m*^*β*^ + 1/(*m*′)^*β*^ for some *β* > 0. In Fig 3, we show the numerical solution to the coagulation equation for both the exponentially decaying kernel (*A* = exp(−*m*′) + exp(−*m*)) and for the constant kernel (*A* = 1). We see that, for *t* < 1, the difference between the two kernels is small. Since the Pareto kernel is in between these two extremes of exponential decay and a constant, we conclude that basing our calculations on the exponential kernel is justified. In section 4, we have also appealed to our intuition that the probability of an M&A increases as a function of the difference in company size. Fig 3 addresses this idea by showing a third kernel (*A* = exp(−1/|*m* − *m*′|)). We can see clearly that our results are not affected by this assumption, at least in the limits of heavy tailed initial distributions and small times (*t* < 1).

To simplify the Laplace transform, we have worked with the fractional exponential distribution with asymptotic power law tail. However, the literature suggests that the lognormal is often also a good approximation of firm size distribution. Empirically, as already mentioned, it can be difficult to distinguish the two. To understand this, note that one can write the log-normal density as

$$\begin{array}{c} p\left(m,t=0\right)=\frac{1}{\sqrt{2\pi \sigma^{2}}}\frac{1}{m}\exp(-\frac{\log^{2}\left(m/m_{0}\right)}{2\sigma^{2}})=\frac{1}{\sqrt{2\pi \sigma^{2}}m_{0}}{\left(m/m_{0}\right)}^{-1-\mu (m)}\propto m^{-1-\mu (m)} \end{array}$$ (25)

with $\mu \left(m\right)=\frac{1}{2\sigma^{2}}\log(\frac{m}{m_{0}})$. The second equality in Eq (25) follows because $e^{{\left(\log m\right)}^{2}}=m^{\log m}$ (cf. section 4.1.3 in [10]). Since *μ*(*m*) is a slowly varying function of *m*, the log-normal distribution can be mistaken for an apparent power law with an exponent *μ* that is gradually changing. Despite this apparent similarity, the log-normal is very different from a Pareto. According to the Pickands-Balkema-de Haan theorem of extreme value theory [53], the log-normal law belongs to the domain of attraction of the Gumbel distribution for the distribution of maxima, and the Gumbel distribution has an exponential tail. For the log-normal, all moments exist and we would expect that the coagulation equation shows qualitatively different behavior for those two initial distributions (power law versus log-normal). Indeed, Menon and Pego [54, 55] show that (for some specific kernels) the solution of the coagulation equation depends in a subtle way on the number of finite moments of the initial distribution. Nevertheless, as is confirmed in Fig 3, these differences are not visible for small times over the considered range of firm sizes. All our theoretical statements seem to apply equally well for other heavy-tailed initial distributions.

## 7 Conclusions & outlook

We have examined the effect of mergers and acquisitions on time evolution of firm size distributions. Specifically, we have described the effect of M&A in terms of a partial integro-differential equation, also known in the literature as the coagulation equation. This approach allowed us to account for the inverse relation of firm size and the probability of being acquired. While the resulting coagulation equation cannot be solved analytically in closed form, we have derived a first order solution that can explain empirical observations appearing counterintuitive at first sight, namely that mergers and acquisitions develop a significant influence on the firm size distribution only over time scales much longer than a few decades. This explains why M&A has apparently little impact on the firm size distributions in existing data sets. Thus, observations of M&A events over much longer time scales are required in order to see a significant deviation from a heavy tailed firm size distribution. Precise numerical solutions have confirmed the validity of our approximations. Hence, we conclude that the coagulation equation is an adequate tool to capture the dynamics of merger and acquisitions on the firm size distribution.

We have made two assumptions that could be relaxed. First, we considered the situation where the initial distribution is already heavy tailed. Implicitly, this takes into account the aggregate effect of internal growth, firm births and bankruptcies. These three mechanisms are known to generically create power law distributions of firm sizes [20, 21]. We also note that the effect of mergers and acquisitions was quite negligible at the early stage of a developing economy, as illustrated for the US. Looking at Fig 1 in [28], one sees that M&A have become popular significantly after Gibrat’s [8] observation of proportional growth. However, it may be that the asymptotic long time behavior of the firm size distribution is strongly dependent on the initial distribution, a problem that is in full generality still under investigation [46]. It is then interesting to ask whether mergers and acquisitions can significantly impact the firm size landscape of a developing economy.

Second, we have only considered M&As and neglected other firm size dynamics such as internal growth, firm creation, firm exits or sudden bankruptcy. Adding spin-off dynamics to the coagulation equation leads to the more general population balance Eq (2). In contrast to pure coagulation, here equilibrium states are possible by offsetting the M&As with spin-offs and creation of new firms. An interesting question is then whether such a balance condition is met in real economies, or whether external growth dynamics acts as a driver towards out-of-equilibrium dynamics. Investigation of such questions is part of future research.
